# Motion of charged particles in bright squeezed vacuum

**DOI:** 10.1038/s41377-024-01381-w

**Published:** 2024-02-01

**Authors:** Matan Even Tzur, Oren Cohen

**Affiliations:** Solid State Institute and Physics Department, Technion-Israel Institute of Technology, Haifa, 3200003 Israel

**Keywords:** High-harmonic generation, Ultrafast photonics, Quantum optics

## Abstract

The motion of laser-driven electrons quivers with an average energy termed pondermotive energy. We explore electron dynamics driven by bright squeezed vacuum (BSV), finding that BSV induces width oscillations, akin to electron quivering in laser light, with an equivalent ponderomotive energy. We identify closed and open trajectories of the electronic width that are associated with high harmonic generation and above-threshold ionization, respectively, similarly to trajectories of the electron position when its motion is driven by coherent light. In the case of bound electrons, the width oscillations may lead to ionization with noisy sub-cycle structure. Our results are foundational for strong-field and free-electron quantum optics, as they shed light on ionization, high harmonic generation, and nonlinear Compton scattering in BSV.

## Introduction

The ponderomotive energy scale *U*_p_ is the cycle averaged energy of a charged particle interacting with a classical, monochromatic, linearly polarized, electric field. It is a key figure of merit in the theory of high-field ionization^1,2^, high harmonic generation^3,4^, and plasma physics^5,6^. Compared to the natural energy scales of a system at hand, the scale of *U*_p_ determines the transition between regimes in light matter interactions. A prominent example is the transition between the multi-photon and tunnel ionization regimes of atoms, which is determined by the Keldysh parameter^1^ $\gamma =\sqrt{{I}_{{\rm{p}}}/2{U}_{{\rm{p}}}}$, in which *I*_p_ is the ionization potential of an atom. *U*_p_ is given by the famous formula (atomic units throughout):

1 $${U}_{{\rm{p}}}^{\left({\rm{c}}\right)}={e}^{2}{E}_{{\rm{a}}}^{2}/4m{\omega }_{{\rm{p}}}^{2}$$

in which *e* is the electron charge, *E*_*a*_ is the peak amplitude of a monochromatic & linearly polarized electric field of frequency *ω*_p_, and *m* is the mass of the electron. A superscript (c) was added to indicate that this ponderomotive energy is ‘classical’, in the sense that it corresponds to the energy of a classical electron in a classical field, as derived by Newton’s equations of motion.

The ponderomotive energy scale is associated with *quiver motion*. A quivering particle oscillates back and forth in space at the frequency of a driving laser, following a trajectory prescribed by the driving laser field. While almost interchangeable with light-induced motion, any sinusoidally oscillating force will induce a quiver motion with an average ponderomotive energy. In the case of (classical) electromagnetic waves & charged particles, this force is the Lorentz force. Notably, if light carries a vanishing coherent electric field amplitude *E*_a_ = 0, the sinusoidally oscillating Lorentz force vanishes, and Eq. (1) yields ${U}_{{\rm{p}}}^{\left({\rm{c}}\right)}=0$ (i.e., the particle stands still).

At the same time, there are many indications that coherent motion of matter may be induced even by quantum fields carrying a vanishing electric field amplitude. A recent prominent example in this context is the interaction of bright squeezed vacuum (BSV) with various phases of matter, in different regimes^7^. Perturbative nonlinear optical processes^8,9^ and photoionization^10^ driven by BSV were already observed experimentally. Remarkably, BSV has been shown to significantly boost the efficiency of these processes by orders of magnitude, indicating a significant potential for technological impact, e.g., in miniaturization of harmonic generation and ultrafast photoelectron sources.

In the contexts of high harmonic generation^11,12^ and nonlinear Compton scattering^13^, a BSV drive was similarly predicted to results in an enhanced efficiency and broader emitted spectrum. Squeezing also provides an additional degree of freedom for attosecond spectroscopy, as it shifts & shapes the emitted attosecond pulses during HHG^12^. Additionally, driving the HHG process by squeezed light is predicted to result in squeezed harmonics^14^, complementing other HHG-based sources of quantum light^15,16^. Squeezed high harmonics eliminate the most fundamental limitation from ultrafast optical interferometry: vacuum fluctuations. State of the art interferometers^17^ already achieve zeptosecond (10^−21^ s) resolution, approaching the yoctosecond-scale (10^−24^ s) limit enforced by vacuum fluctuations^12^. Yet, as BSV carries a vanishing coherent electric field amplitude, exhibiting only electric field *fluctuations*, the foundational concepts of quiver motion & ponderomotive energy are inapplicable, limiting our understanding of BSV-driven sources of extreme light^11,13^ & fast electrons^10^.

Here we generalize the concepts of quiver motion and its associated ponderomotive energy for charged particles in quantum light fields, focusing on the multi-mode squeezed vacuum state. More generally, we explore *sub-cycle motion* of free & bound particles driven by bright squeezed vacuum. We calculate numerically the motion of a free electron driven by a bright squeezed vacuum field. We find that free electrons in bright squeezed vacuum undergo width oscillations, i.e., coherent stretching and squeezing in real space. We show numerically and analytically that the cycle-averaged energy associated with these width oscillations, namely, the quantum ponderomotive energy ${U}_{{\rm{p}}}^{({\rm{q}})}$, is equal to the classical ponderomotive energy ${U}_{{\rm{p}}}^{\left({\rm{c}}\right)}$ for equally intense BSV and coherent fields. Furthermore, we found that the width oscillations exhibit closed and open trajectories that contribute to HHG and ATI, respectively, similarly to displacement trajectories when the motion is driven by coherent light. In the case of bound electrons, we find that such width oscillations may be violent enough to induce ionization (and recombination), which follow noisy sub-cycle dynamics. Our results are foundational to extreme nonlinear quantum optics, as they provide insight to the underlying mechanisms of nonlinear Compton scattering^13^, HHG^11,12^, and ionization^10^ when they are driven by squeezed vacuum.

## Results

### Free electron width oscillations and their ponderomotive energy

**Fig. 1:**
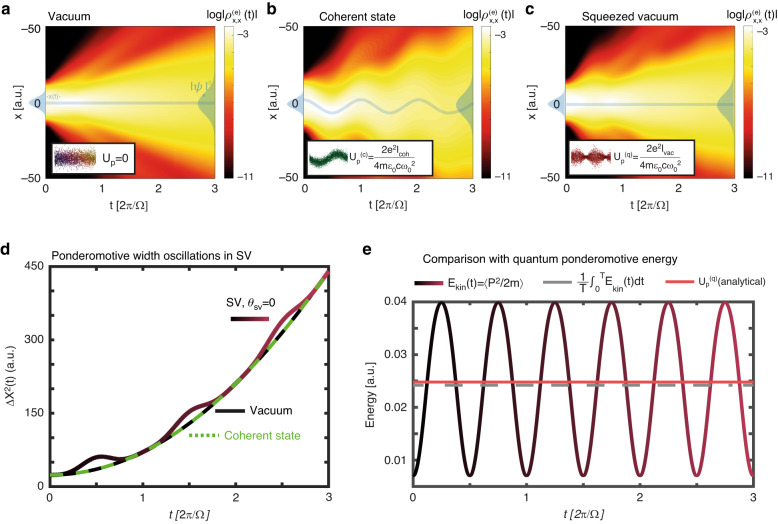
Numerical time evolution of a free electron in quantum fields. **a–c** Electron density ${\rho }_{x,{x}}^{\left(e\right)}\left(t\right)$ for an electron coupled to (**a**) quantized electromagnetic vacuum (**b**) a coherent state (**c**) a single mode of squeezed vacuum. **d** Wave packet width as a function of time for the three cases plotted in (**a**–**c**). **e** time dependent expectation value of the kinetic energy. The time averaged energy (gray, dashed line) matches the analytically derived ponderomotive energy (pink, solid line)

We begin by calculating numerically the motion of a free electron placed in a bright-squeezed vacuum field. We performed three time evolution calculations for an electron that initially occupies a gaussian wavepacket in 1D real space $\left|{\rm{g}}\right\rangle \propto \exp \left(-x/4{\sigma }_{0}^{2}\right)$ and interacts with: (i) a single mode of EM vacuum at frequency Ω, $\left|{0}_{\Omega }\right\rangle$, (ii) a coherent state $\hat{D}\left(\alpha \right)|{0}_{\Omega }\rangle$, and (iii) a single mode of squeezed vacuum $\hat{S}\left(r\right)\left|{0}_{\Omega }\right\rangle$. Here, $\hat{D}\left(\alpha \right)$ and $\hat{S}\left(r\right)$ are coherent shift and squeezing operators for the temporal mode Ω, respectively^18^. Time evolution of the initial light-matter state under the Hamiltonian $\hat{H}={\hat{p}}^{2}/2m+\hat{x}\cdot {\hat{E}}_{\Omega }\left(t\right)$ is implemented through the (*t,t’*) method^19^ (SI section II). Here, $\hat{x}$ and $\hat{p}$ are the electron position and momentum operators respectively, and ${\hat{E}}_{\Omega }\left(t\right)\equiv \sqrt{\hslash \Omega /2{\epsilon }_{0}V}\left({\hat{a}}_{\Omega }{e}^{-i\varOmega t}+{{\hat{a}}^{\dagger }}_{\varOmega }{e}^{i\varOmega t}\right)$ is the electric field operator of the Ω mode (pump), and *V* is the quantization volume. We set Ω = 0.11 a.u. and $\sqrt{\hslash \Omega /2{\epsilon }_{0}V}=1\times {10}^{-8}$ a.u. to match the experimentally observed value of vacuum fluctuations of the order of 50 V cm^−1^ ^20^. These calculations yield the time-dependent light matter state $\left|\Psi \left(t\right)\right\rangle$ for each initial driving light state. Finally, a partial trace on the photonic degrees of freedom is implemented, resulting in the reduced density matrix of the electron ${\rho }_{x,{x}^{{\prime} }}^{\left(e\right)}\left(t\right)$, whose diagonal is the real-space electron density, (equivalent to ${\left|{\psi }_{e}\left(x,t\right)\right|}^{2}$ for pure states). Figure 1a–c present the density ${\rho }_{x,{x}}^{\left(e\right)}\left(t\right)$ for the three examined cases. Notably, while the coherent state induces a quivering displacement motion ${\left\langle \hat{x}\left(t\right)\right\rangle }_{{\rm{CS}}}\,\ne \,0$, that matches the Newtonian trajectory of a charged particle, the squeezed vacuum state results in a vanishing displacement ${\left\langle \hat{x}\left(t\right)\right\rangle }_{{\rm{SV}}}=0$. Examining the width of the wavepackets $\Delta {X}^{2}=\left\langle {\hat{x}}^{2}\left(t\right)\right\rangle -{\left\langle \hat{x}\left(t\right)\right\rangle }^{2}$, we find that for the EM vacuum & coherent state fields, the Gaussian wavepacket expands according to the analytical formula $\Delta {X}^{2}\left(t\right)={\sigma }_{0}^{2}\left(1+{t}^{2}/4{\sigma }_{0}^{4}\right)$ (Fig. 1d). In contrast, the width of the SV driven electron is periodically modulated, exhibiting coherent stretching & squeezing dynamics in real space, superimposed on the quadratic expansion of the Gaussian wavepacket. Figure 1e presents the time dependent kinetic energy of the electron obtained from the numerical calculation, *E*_kin_ (*t*). The cycle-average of *E*_kin_ (*t*) is the quantum-optical generalization of the ponderomotive energy, ${U}_{{\rm{p}}}^{\left({\rm{q}}\right)}=\frac{1}{T}{\int }_{0}^{T}{E}_{{\rm{kin}}}\left(t\right){dt}$. The numerical calculation reveals that ${U}_{{\rm{p}}}^{\left({\rm{q}}\right)}$ is exactly equal to the classical ponderomotive energy imposed by an equally intense coherent state:

2 $${U}_{{\rm{p}}}^{\left({\rm{q}}\right)}=\frac{2{e}^{2}}{m{\epsilon }_{0}c}\,\frac{{I}_{{\rm{vac}}}}{4{\Omega }^{2}}$$

Here, *m,e* are the mass and charge of the electron, *ϵ*_0_ vacuum permittivity, *c* speed of light. ${I}_{{\rm{vac}}}\equiv c\hslash \Omega {N}_{{\rm{SV}}}/V$ is the intensity of a squeezed vacuum beam with ${N}_{{\rm{SV}}}\equiv {\sinh }^{2}\left(r\right)$ photons in a quantization volume *V*, and a frequency Ω. The number of photons in a squeezed vacuum state is given by ${N}_{{\rm{SV}}}={\sinh }^{2}\left(r\right)$ where *r* is the dimensionless squeezing parameter^21^. In section I of the SI, we derive Eq. (2) analytically using 2nd order perturbation theory, and generalize it to multi-mode squeezed light, accounting for various forms of squeezed light such as polarization squeezed light and more. For multi-mode squeezed light, the quantum ponderomotive energy is given by:

3 $${U}_{{\rm{p}}}^{\left({\rm{q}},{\rm{multimode}}\right)}\equiv \frac{{{\hslash }}{e}^{2}}{4{\pi }^{2}m}\,\sum _{j}\int {d}^{3}{{\boldsymbol{k}}}_{j}\frac{{|\nu \left({{\boldsymbol{k}}}_{j}\right)|}^{2}}{{\omega }_{{\boldsymbol{k}}j}}$$

Where |*ν*(***k***_*j*_)|^2^ is the number of photons in the ***k***_***j***_ mode in the multi-mode squeezed vacuum state. That is, the ponderomotive energy in multi-mode squeezed vacuum depends only on the intensity spectrum of the pump. Notably, these formulas show that the classical formula for ponderomotive energy, derived through Newton’s equations of motion, applies to any form of multi-mode squeezed light, despite the fact the underlying electron motion is very different from the classical case. Furthermore, as a corollary of our calculation, we find that the electron experiences a mass renormalization contingent upon vacuum squeezing, and that it is miniscule for non-relativistic velocities (Eq. I.24 in the SI).

### Closed & open trajectories of the width

**Fig. 2:**
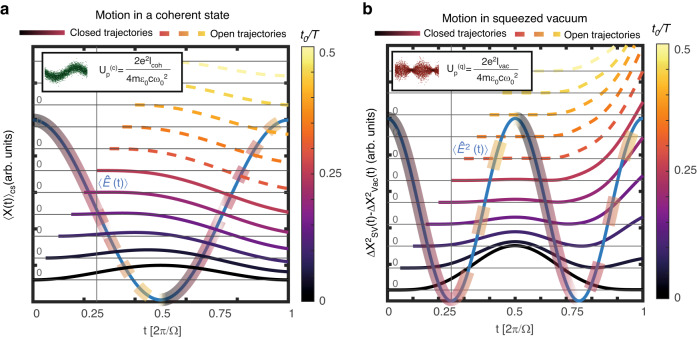
Closed and open trajectories of free electrons. **a** The displacement of a free electron coupled to a coherent state for various initial moments of the interaction *t*_0_, calculated using Newton’s equations of motion. Each trajectory corresponds to a different initial time *t*_0_, with the trajectories offset for visibility and their respective *y*-axes marked with a horizontal gray line. The electric field is depicted in blue. **b** The excess width of a free electron driven by squeezed vacuum for various initial moments, calculated by numerical time evolution of a Gaussian wavepacket driven by squeezed vacuum (SI section II). Similarly to (**a**), each trajectory corresponds to a different initial time *t*_0_, with the trajectories offset for visibility and their respective *y*-axes marked with a horizontal gray line. The variance of the electric field is depicted in blue. Both observables (**a**, **b**) exhibits “closed” trajectories for initial times *t*_0_ between 0 and 0.25 T (solid lines), and open trajectories between 0.25 T and 0.5 T (dashed lines)

When a charged particle is released in a classical electromagnetic field ∝ cos (*ωt*) at a position *x* = 0 and time *t* *=* *t*_0_, it may exhibit either closed or open motion (Fig. 2a). If the electron started its motion after the peak (node) of the field, i.e., $0 \,<\, {t}_{0}/T < 0.25$ ($0.25 < {t}_{0}/T \,< \,0.5$), it will (not) revisit *x* = 0 at a later time, resulting in a closed (open) trajectory. The notion of closed & open trajectories is central to the description HHG and ATI when driven by a coherent state. For instance, if the electron starts its motion at the ionization time *t*_0_, only ionization times in the range $0 < {t}_{0}/T < 0.25$ contribute to HHG and re-scattering ATI, because they result in closed trajectories. An analogous phenomenon exists in the context of the motion of charged particles in squeezed vacuum (Fig. 2b). A free electron that begins its motion after the anti-squeezed peak of the SV field variance will exhibit a closed trajectory in the sense that its width will revisit the free propagation width at a later time (at least once). Likewise, if the electron is released to motion right after the maximal squeezing of the field $0.25 < {t}_{0}/T < 0.5$ it will exhibit an open trajectory, i.e., it will always be wider than the free propagation width. In Fig. 2b, we numerically observe the dynamics of the excess width $\Delta {{\rm{X}}}_{{\rm{SV}}}^{2}({\rm{t}})-\Delta {{\rm{X}}}_{{\rm{Vac}}}^{2}({\rm{t}})$ for a Gaussian electron wavepacket driven by a single mode of squeezed vacuum. This is computed by numerically time-evolving the wavepacket from an initial time *t*_0_, calculating its width $\Delta {{\rm{X}}}_{{\rm{SV}}}^{2}({\rm{t}})$ as a function of time, and then subtracting the width obtained for zero squeezing, $\Delta {{\rm{X}}}_{{\rm{Vac}}}^{2}({\rm{t}})$.

To understand this motion, we consider the perturbative formula for the reduced density matrix of the electron *ρ*^(*e*)^, after interaction with the squeezed vacuum. The formula, derived using a quasi-probability distribution approach, reads^12^:

4 $${\rho }^{\left(e\right)}\approx \frac{1}{\sqrt{2\pi }\left|{E}_{{\rm{vac}}}\right|}\int d{E}_{\alpha }{e}^{-\frac{{\left|{E}_{\alpha }\right|}^{2}}{2{\left|{E}_{{\rm{vac}}}\right|}^{2}}}\,\left|{\phi }_{{E}_{\alpha }}\left(t\right)\right\rangle \left\langle {\phi }_{{E}_{\alpha }}\left(t\right)\right|$$

5 $${I}_{{vac}}\equiv \frac{1}{2}{\epsilon }_{0}c{\left|{E}_{{\rm{vac}}}\right|}^{2}$$

where *I*_vac_ is the intensity of the squeezed vacuum beam, and |*E*_vac_| is the electric field amplitude of an equally intense Glauber coherent state. It is also approximately the amplitude of electric field fluctuations in the anti-squeezed quadrature of the pump. The wavefunction $\left|{\phi }_{{E}_{\alpha }}\left(t\right)\right\rangle$ solves the following time dependent Schrödinger equation of a free electron in a classical electric field ${E}_{\alpha }=\left\langle \alpha \left|\hat{E}\left(t\right)\right|\alpha \right\rangle$

6 $$i{\rm{\hslash }}\frac{\partial \left|{\phi }_{{E}_{\alpha }}\left(t\right)\right\rangle }{\partial t}=\left[-\frac{1}{2m}{\nabla }^{2}-{ex}\cdot {E}_{\alpha }\cos \left(\varOmega t\right)\right]\left|{\phi }_{{E}_{\alpha }}\left(t\right)\right\rangle$$

With the initial condition $\left|{\phi }_{\alpha }\left(t=0\right)\right\rangle =\left|g\right\rangle$. According to Eq. (4,  6), the width of the wavepacket is given by

7 $$\Delta {X}^{2}(t)\approx \mathop{\underbrace{{\sigma }_{0}^{2}+\frac{{t}^{2}}{4{\sigma }_{0}^{2}}}}\limits_{{\rm{vacuum}}\,{\rm{expansion}}}+\mathop{\underbrace{{\langle {x}_{{E}_{{\rm{vac}}}}(t)\rangle }^{2}}}\limits_{{\rm{Width}}\,{\rm{oscillations}}}$$

Where $\left\langle {x}_{{E}_{{\rm{vac}}}}\left(t\right)\right\rangle$ is the classical displacement of $\left|{\phi }_{{E}_{\alpha }}\left(t\right)\right\rangle$ with ${E}_{\alpha }={E}_{{\rm{vac}}}$, i.e., the displacement an equally intense coherent state would impose. Because $\left\langle {x}_{{E}_{{\rm{vac}}}}\left(t\right)\right\rangle$ results in closed trajectories between $0 \,<\, {t}_{0} \,< \,0.25T$, so does $\Delta {X}^{2}\left(t\right)$. For $0.25T < {t}_{0} < 0.5T$, the displacement $\left\langle {x}_{{E}_{{\rm{vac}}}}\left(t\right)\right\rangle$ represents an open trajectory, therefore $\Delta {X}^{2}\left(t\right)$ does not cross the vacuum expansion line. Similarly to the case of a coherent state driver, we anticipate that only closed trajectories of the width contribute to HHG and re-scattering ATI, when they are driven by squeezed vacuum. Our reasoning is as follows: Eq. (7), that describes the width trajectories $\left\langle {x}^{2}\left(t\right)\right\rangle$, shows that the width trajectories are obtained from an average of displacement trajectories, each corresponding to a coherently driven electronic state $\left|{\phi }_{{E}_{\alpha }}\left(t\right)\right\rangle$ (Eq. (4)). When the width trajectory is closed, the displacement $\left\langle {x}_{{E}_{\alpha }}\left(t\right)\right\rangle$ of each individual $\left|{\phi }_{{E}_{\alpha }}\left(t\right)\right\rangle$ in the average exhibits a closed trajectory, which leads to high harmonics & re-scattering ATI. Similarly, for open width trajectories, the individual constituents of the superposition do not follow closed displacement trajectories, and therefore do not contribute to HHG & re-scattering ATI.

### Sub-cycle dynamics of photoionization driven by bright-squeezed vacuum

**Fig. 3:**
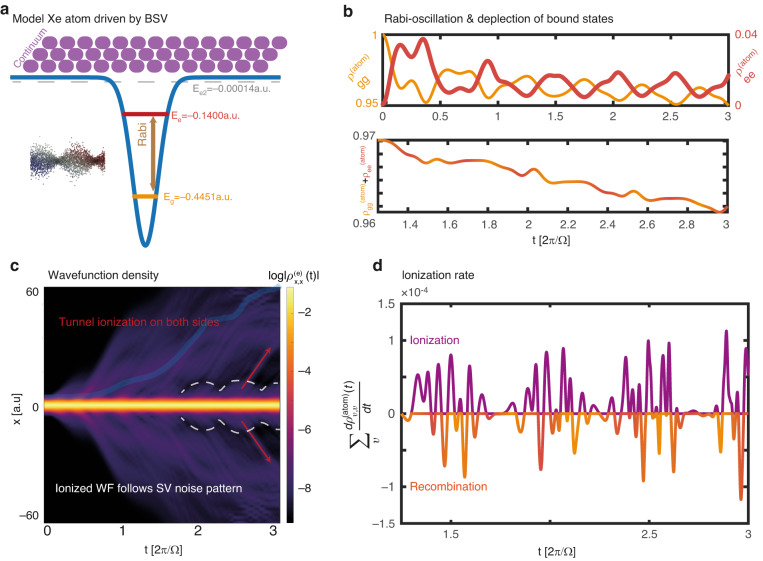
Sub-cycle dynamics of bound electrons driven by BSV. (**a**) illustration of a model Xe atom interacting with a squeezed vacuum field (**b**) populations of the ground and 1^st^ excited state as a function of time. The states undergo Rabi oscillations, while steadily depleting as a function of time due to ionization. **c** Electron wavefunction density in real space as a function of time ${\rho }_{x,{x}}^{\left(e\right)}\left(t\right)$ (**d**) time derivation of total continuum state populations, indicative of the ionization rate as a function of time. The ionization occurs in noisy bursts that are in phase with the squeezed vacuum noise

Next, we explore the dynamics of a bound electron in bright squeezed vacuum by adding a model Xe atomic potential to the numerical calculation^22^. This model atom supports two bound states with energies ${E}_{g}=0.44$ a.u. and ${E}_{e}=-0.14$ a.u., as well as a third weakly bound state with an energy ~0.00014 a.u. and a continuum. We calculate the time evolution of the ground state of this atom ${\left|{\rm{g}}.{\rm{s}}.\right\rangle }_{{\rm{Xe}}}$ when it is driven by BSV and coupled to one quantized radiation mode (SI section II). Again, we obtain the time-dependent light-matter state $\hat{\rho }\left(t\right)$ and perform a partial trace on the photonic degrees of freedom to obtain the reduced density matrix of the atom *ρ*^(atom)^. Figure 3(b) presents the populations of the ground and 1^st^ excited states ${\rho }_{{\rm{gg}}}^{\left({\rm{atom}}\right)}$ and ${\rho }_{{\rm{ee}}}^{\left({\rm{atom}}\right)}$, showing Rabi-like oscillations between these levels. Their total population is nearly flat with a negative slope, with the slope being the time averaged ionization rate. Fourier analysis of the atomic inversion (SI section III) ${\rho }_{{\rm{ee}}}^{\left({\rm{atom}}\right)}\left(t\right)-{\rho }_{{\rm{gg}}}^{\left({\rm{atom}}\right)}\left(t\right)$ shows that it consists of two distinct spectral peaks. The dominant of these peaks corresponds to the detuning frequency ${\delta }_{12}={E}_{{\rm{e}}}-{E}_{{\rm{g}}}-\Omega =0.1951$ a.u., typical of Rabi-oscillations in a highly detuned regime^18^. The 2nd frequency of oscillations is the transition frequency between the ground and 2nd excited state ${E}_{{\rm{e}}2}-{E}_{{\rm{g}}}=0.44$ a.u., which is also approximately the ionization potential, and is resonant with the 4th harmonic of the pump.

Figure 3c shows the time-dependent density of the electron in real space. It is evident that the electron undergoes ionization during the interaction, but unlike ionization in a coherent state of light, it is symmetric to both sides of the atomic potential well. Time-frequency analysis of the wavepacket width reveals that the high-frequency components of the dynamics occur within half-cycle temporal windows, with a chirp corresponding to semi-classical recombination times (SI section III). Figure 3d shows the time derivative of the total population of all continuum states, indicative of the rate of ionization. We observe that this quantity exhibits rapid oscillations between positive and negative rates, i.e., the system is rapidly switching from net ionization to net recombination. The amplitude of the oscillations is in phase with the amplitude of electric field fluctuations of the squeezed vacuum. We anticipate light emission (and particularly HHG) to occur during the recombination bursts, as the electron must release its kinetic energy in the form of radiation.

## Discussion

### Proposed experiment

**Fig. 4:**
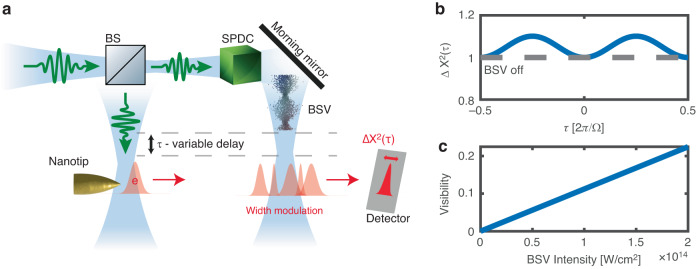
Suggested experiment. **a** Suggested experimental setup for measuring the width dynamics of the electronic wave-packet in BSV. An ultrashort photoelectron wavepacket is generated by nanotip photoionization. The width of the photoelectron is subsequently modulated by interaction with a BSV pulse. The relative delay between the ionizing and modulating pulses is scanned using a moving mirror, and the width of the photo-electron wavepacket is measured by a detector as a function of the relative delay. **b** The width of the photoelectron modulated by a BSV of intensity 3.2e13 W cm^−2^ and frequency $\varOmega =0.11$ a.u. The width oscillates sinusoidally with the relative delay, with a frequency of 2Ω, where Ω is the frequency of the pump. The minimum width of the modulated electron is predicted to coalesce with the width of the photoelectron when the BSV arm is blocked (here set to 1 a.u.^2^). **c** The visibility (contrast) of the modulation is predicted to increase linearly with the BSV intensity

Figure 4 illustrates a proposed experiment for observing the modulation of the electron wavepacket width by BSV. A beam splitter splits an ultrashort laser pulse into two arms. The first arm ionizes a nanotip, generating an ultrashort propagating electron wavepacket^23^. In the second arm, a high-gain spontaneous parametric down-conversion process in a *χ*^(2)^ nonlinear crystal creates broadband squeezed vacuum (BSV) light^24^. This BSV light is filtered to obtain a single spatial and temporal mode^24^. Subsequently, the BSV pulse is reflected off a mirror, and directed towards the electron to modulate its width. Regardless of the BSV pulse’s duration, the width undergoes sinusoidal modulation as a function of the relative delay between the ionizing coherent pulse and the modulating BSV pulse (Eq. (7) and Fig. 4b). This relative delay is controlled by translating the mirror using a translation stage. The minimum width of the electron is predicted to coalesce with the width observed when the BSV arm is blocked. This minimum width corresponds to the electron entering the BSV field at the peak of the field fluctuations, as illustrated in Fig. 2b. Additionally, the contrast of this modulation increases linearly with the intensity of the BSV, as depicted in Fig. 4c.

## Conclusion

To summarize, we have generalized the classical notions of quiver motion and ponderomotive energy to a quantum optical context. We have shown theoretically that the motion of a free electron in squeezed vacuum consists of periodic stretching and squeezing of its wavefunction in real space and discovered that it exhibits open and close trajectories, in a similar fashion to displacement trajectories of electrons driven by coherent light. For the case of a bound electron, our results shed light on the underlying dynamics associated with high harmonic generation driven by BSV and resolve sub-cycle features of ionization. Additionally, we found that the energy of an electron interacting with a generalized multi-mode squeezed vacuum field is exactly equal to the classical ponderomotive energy (derived by Newton’s equations of motion when the electron is driven by a classical field). Our treatment holds for any mutli-mode form of squeezed light, for example, bi-chromatic two-mode squeezed vacuum^25^, as well as for polarization squeezed light^26^. Looking forward, we expect our results to be directly applicable to nonlinear Compton scattering^13^ driven by BSV and free electron shaping by quantum light^27,28^, as both interactions are concerned with a free electron placed in a squeezed vacuum field. Additionally, as the presented results generalize the standard building blocks (*U*_p_ and quiver motion) of strong-field physics to the quantum optical regime, we believe our results will play a foundational role in the emerging field of quantum-optical strong-field physics, which ranges from quantum information processing^15^, explorations of light-matter entanglement^29–34^, and more^35–38^.

### Supplementary information

Supplementary Information for “Motion of charged particles in bright squeezed vacuum”
