# Supplementary material for: Motion of charged particles in bright squeezed vacuum
Source: Light Sci Appl. 2024 Feb 1;13:41. doi: 10.1038/s41377-024-01381-w (PMC10834546; doi:10.1038/s41377-024-01381-w)
Supplement: Supplementary file 1 — Supplementary Information for “Motion of charged particles in bright squeezed vacuum” [file 41377_2024_1381_MOESM1_ESM.pdf]

Supplementary Information for "Motion of charged particles in bright squeezed vacuum"

Matan Even Tzur and Oren Cohen

Solid State Institute and Physics Department, Technion-Israel Institute of Technology, Haifa 3200003, Israel

This supplementary information file contains additional details about the derivations and numerical calculations presented in the main text. In section I we present the detailed derivation of the ponderomotive energy shift and squeezing dependent mass renormalization presented in the main text. In section II we present the numerical methodology used in the time evolution calculations presented in the main text. In section III we present Fourier analysis of the dynamics of an atom driven by bright squeezed vacuum.

I. Detailed derivation of the ponderomotive energy and a squeezing-dependent mass shift

In this section, we perform a detailed derivation of the ponderomotive energy of a free electron in multimode squeezed vacuum and derive a squeezing dependent mass renormalization factor. We begin by considering the canonically quantized Hamiltonian of the light matter system.

$$H = H_0 + V_1 + V_2 \tag{I.1}$$

$$H_0 = -\frac{\hbar^2}{2m}\int \psi^\dagger \nabla^2 \psi \, d^3\boldsymbol{x} + \frac{1}{8\pi}\int (E_\perp^2 + H^2) d^3\boldsymbol{x}$$

$$V_1 = \frac{e}{mc}\int \psi^\dagger \boldsymbol{A}_\perp \cdot \frac{\hbar}{i}\nabla\psi \, d^3\boldsymbol{x}$$

$$V_2 = \frac{e^2}{2mc^2}\int \psi^\dagger \boldsymbol{A}_\perp^2 \psi d^3\boldsymbol{x}$$

Here, $|\psi(\boldsymbol{x})\rangle$ is the wavefunction of the electron, $E_\perp, A_\perp$ are the transverse parts of the electric field & vector potential field (coulomb gauge), and H is the magnetic field. e electron charge, c speed of light, and m is the **bare undressed mass of the electron.** Upon canonical quantization in volume V we have

$$\psi(\boldsymbol{x}) = \frac{1}{\sqrt{V}}\sum \hat{c}_{\boldsymbol{p}} e^{(i/\hbar)\boldsymbol{p}\cdot\boldsymbol{x}} \tag{I.2}$$

$$A_\perp(\boldsymbol{x}) = \sum \sqrt{\frac{2\pi\hbar c^2}{V\omega_k}} \left(\boldsymbol{e}_{\boldsymbol{k}j}\hat{a}_{\boldsymbol{k}j} e^{i\boldsymbol{k}\cdot\boldsymbol{x}} + \boldsymbol{e}_{\boldsymbol{k}j}^* \hat{a}_{\boldsymbol{k}j}^\dagger e^{-i\boldsymbol{k}\cdot\boldsymbol{x}}\right)$$

$$\boldsymbol{E}(\boldsymbol{x},t) = -\frac{1}{c}\dot{A} = i\sum_{\boldsymbol{k}j} k\left\{\boldsymbol{e}_{\boldsymbol{k}j}A_{\boldsymbol{k}j}e^{-i\omega_k t}e^{i\boldsymbol{k}\cdot\boldsymbol{x}} - \boldsymbol{e}^*_{\boldsymbol{k}j}A^*_{\boldsymbol{k}j}e^{i\omega_k t}e^{-i\boldsymbol{k}\cdot\boldsymbol{x}}\right\}$$

$$\boldsymbol{H}(\boldsymbol{x},t) = \nabla\times\boldsymbol{A} = i\sum_{\boldsymbol{k}j} k\left\{\boldsymbol{b}_{\boldsymbol{k}j}A_{\boldsymbol{k}j}e^{-i\omega_k t}e^{i\boldsymbol{k}\cdot\boldsymbol{x}} - \boldsymbol{b}^*_{\boldsymbol{k}j}A^*_{\boldsymbol{k}j}e^{i\omega_k t}e^{i\boldsymbol{k}\cdot\boldsymbol{x}}\right\}$$

Here, V is the quantization volume (i.e., modal volume of the electromagnetic field) and periodic boundary conditions are assumed to apply. The following relations follow from the canonical commutation relations:

$$\boldsymbol{b}_{\boldsymbol{k}j} = \hat{\boldsymbol{k}}\times\boldsymbol{e}_{\boldsymbol{k}j} \tag{I.3}$$

$$\sum_j \left(\boldsymbol{e}_{\boldsymbol{k}j}\right)_m\left(\boldsymbol{e}^*_{\boldsymbol{k}j}\right)_n = \sum_j \left(\boldsymbol{b}_{\boldsymbol{k}j}\right)_m\left(\boldsymbol{b}^*_{\boldsymbol{k}j}\right)_n = \delta_{mn} - \hat{k}_m\hat{k}_n$$

$$\sum_j \left(\boldsymbol{e}_{\boldsymbol{k}j}\right)_m\left(\boldsymbol{b}^*_{\boldsymbol{k}j}\right)_n = e_{mnl}\hat{k}_l$$

Here, e_{mnl} is the completely antisymmetric Levi–Civita pseudo tensor. Rewriting the Hamiltonian in terms of annihilation and creation operators, we have

$$H_0 = \sum\frac{1}{2m}\boldsymbol{p}^2c^\dagger_{\boldsymbol{p}}c_{\boldsymbol{p}} + \sum\hbar\omega_k\left(a^\dagger_{\boldsymbol{k}j}a_{\boldsymbol{k}j} + \frac{1}{2}\right) \tag{I.4}$$

$$V_1 = \frac{e}{m}\sum_{\boldsymbol{p},k_j}\sqrt{\frac{2\pi\hbar}{V\omega_k}}\left(\boldsymbol{e}_{\boldsymbol{k}j}\cdot\boldsymbol{p}c^\dagger_{\boldsymbol{p}+\hbar\boldsymbol{k}}c_{\boldsymbol{p}}a_{\boldsymbol{k}j} + h.c.\right)$$

$$V_2 = \frac{\pi\hbar e^2}{mV}\sum_{\boldsymbol{pp'}}\sum_{\boldsymbol{kk'},jj'}\frac{1}{\sqrt{\omega_k\omega_{k'}}}$$

$$\times\left(\boldsymbol{e}_{\boldsymbol{k}j}\cdot\boldsymbol{e}_{\boldsymbol{k'}j'}c^\dagger_{\boldsymbol{p}}c_{\boldsymbol{p'}}a_{\boldsymbol{k}j}a_{\boldsymbol{k'}j'}\delta_{\boldsymbol{p}-\boldsymbol{p'},\hbar(\boldsymbol{k}+\boldsymbol{k'})} + \boldsymbol{e}_{\boldsymbol{k}j}\right.$$

$$\left.\cdot\,\boldsymbol{e}^*_{\boldsymbol{k'}j'}c^\dagger_{\boldsymbol{p}}c_{\boldsymbol{p'}}a_{\boldsymbol{k}j}a^\dagger_{\boldsymbol{k'}j'}\delta_{\boldsymbol{p}-\boldsymbol{p'},\hbar(\boldsymbol{k}-\boldsymbol{k'})} - \frac{1}{2}c^\dagger_{\boldsymbol{p}}c_{\boldsymbol{p}}\delta_{\boldsymbol{pp'}}\delta_{jj'} + h.c.\right)$$

The terms $\hat{V}_1$ and $\hat{V}_2$ couple the free electron and the vacuum, introducing corrections to the eigenstates of the unperturbed system $\left|\boldsymbol{P},\{n_{\boldsymbol{k}j}\}\right\rangle$, whose energy is

$$E_0\left(\left|\boldsymbol{P},\{n_{\boldsymbol{k}j}\}\right\rangle\right) = \frac{1}{2m}\boldsymbol{P}^2 + \sum_{k_j}\hbar\omega_k\left(n_{\boldsymbol{k}j} + \frac{1}{2}\right) \tag{I.5}$$

To calculate the correction to the unperturbed (semi-classical) state of the electron $|\phi\rangle = \left|\boldsymbol{P},\{0_{\boldsymbol{k},\boldsymbol{j}}\}\right\rangle$, we employ perturbation theory in the coupling coefficient between the electrons and the EM field. The state $\left|\boldsymbol{P},\{0_{\boldsymbol{k},\boldsymbol{j}}\}\right\rangle$ is corrected by the coupling to:

$$
\left|\boldsymbol{P},\{0_{\boldsymbol{k},\boldsymbol{j}}\}\right\rangle' = \left\{\begin{array}{c} 1+\frac{1}{E_0-H_0}(1-P_0)V_1 \\ +\frac{1}{E_0-H_0}(1-P_0)V_2+ \\ \frac{1}{E_0-H_0}(1-P_0)V_1\frac{1}{E_0-H_0}(1-P_0)V_1 \\ -\langle\phi|V_1|\phi\rangle\frac{1}{(E_0-H_0)^2}(1-P_0)V_1 \\ -\frac{1}{2}\langle\phi|V_1\frac{1}{(E_0-H_0)^2}(1-P_0)V_1|\phi\rangle \end{array}\right\}\left|\boldsymbol{P},\{0_{\boldsymbol{k},\boldsymbol{j}}\}\right\rangle \tag{I.6}
$$

Here, $P_0 = |\phi\rangle\langle\phi|$ is a projection operator on the initial state (uncoupled to the vacuum) and E_0 is the unperturbed energy. Notice that we have only included contributions up to e^2 in the wavefunction. The energy of this state is given by (in 2nd order perturbation theory)

$$
E^{(2)} = E_0 + \langle\phi|V_1|\phi\rangle + \langle\phi|V_2|\phi\rangle + \left\langle\phi\left|V_1\frac{1}{E_0-H_0}(1-P_0)\,V_1\right|\phi\right\rangle \tag{I.7}
$$

Free electron in EM vacuum

The first correction term $\langle\phi|V_1|\phi\rangle$ vanishes because it contains no diagonal elements in $\left|\boldsymbol{p},\{n_{\boldsymbol{k},\boldsymbol{j}}\}\right\rangle$ basis. The second term can be trivially shown to be equal to $\frac{\pi\hbar e^2}{mV}\sum_{\boldsymbol{k},j}\frac{1}{\omega_{\boldsymbol{k}j}}$, and is independent of the momentum of the electron. For this reason, it is subtracted from the Hamiltonian and plays no role in the dynamics of the electron. The third and last term is given by

$$
\langle\phi|V_1\frac{1}{E_0-H_0}(1-P_0)V_1|\phi\rangle = -\frac{e^2}{\hbar c^2}\frac{4\hbar}{3\pi}\frac{p^2}{2m^2}\int_0^{\frac{mc}{\hbar}} dk = -\frac{e^2}{\hbar c}\frac{4}{3\pi}\frac{p^2}{2m} \tag{I.8}
$$

Where a Compton cutoff was introduced in accordance with the non-relativistic nature of the calculation. The implies that

$$
\begin{gathered}
\frac{p^2}{2m^*} = \left(1-\frac{e^2}{\hbar c}\frac{4}{3\pi}\right)\frac{p^2}{2m} \\
m^* = \frac{m}{\left(1-\frac{e^2}{\hbar c}\frac{4}{3\pi}\right)} \approx \left(1+\frac{e^2}{\hbar c}\frac{4}{3\pi}\right)m = m + \frac{\delta m}{m}m
\end{gathered} \tag{I.9}
$$

Therefore, $\frac{\delta m}{m^*} \approx \frac{\delta m}{m} = \frac{4\alpha}{3\pi} \approx 0.0031$.

Free electron in squeezed EM vacuum

The electric field is decomposed in quadratures

$$E(t) = \sqrt{\frac{\hbar\Omega^3}{16\pi^3\epsilon_0 c^3}}\,[X_1(t)\cos(\Omega t) + X_2(t)\sin(\Omega t)]$$

$$X_1(t) = \frac{1}{2}\left[a(t)e^{i\Omega t} + a^\dagger(t)e^{-i\Omega t}\right]$$

$$X_2(t) = \frac{1}{2i}\left[a(t)e^{i\Omega t} - a^\dagger(t)e^{-i\Omega t}\right] \tag{I.10}$$

$$a(t) = \int_0^\infty d\omega\widehat{D}(\omega)a(\omega)e^{-i\omega t}$$

Where $D(\omega)$ is the density of states operator. The output of a parametric amplifier may be regarded as squeezed vacuum state, characterized by the functions $\mu(\boldsymbol{k})$, $\nu(\boldsymbol{k})$ and the frequency Ω (carrier frequency). The squeezed vacuum state is given by $\left|0^{(s)}\right\rangle = U|0\rangle$ were

$$U^\dagger a(\omega)U = \mu(\omega)a(\omega) + \nu(\omega)a^\dagger(2\omega_\Omega - \omega)$$

$$U^\dagger a^\dagger(\omega)U = \mu^*(\omega)a^\dagger(\omega) + \nu^*(\omega)a(2\omega_\Omega - \omega)$$

$$|\mu(\omega)| - |\nu(\omega)|^2 = 1$$

The following relations will be useful in the derivation below:

$$\langle a(\omega)a(\omega')\rangle = \mu(\omega)\nu(\omega')\delta(2\omega_\Omega - \omega - \omega')$$

$$\left\langle a(\omega)a^\dagger(\omega')\right\rangle = \mu(\omega)\mu^*(\omega')\delta(\omega - \omega')$$

$$\left\langle a^\dagger(\omega)a(\omega')\right\rangle = \nu(\omega)\nu^*(\omega')\delta(\omega - \omega') \tag{I.11}$$

$$\left\langle a^\dagger(\omega)a^\dagger(\omega')\right\rangle = \nu^*(\omega)\mu^*(\omega')\delta(2\omega_\Omega - \omega - \omega')$$

$$\left\langle a^\dagger(t)a(t)\right\rangle = \frac{1}{\Omega^3}\int_0^\infty d\omega\omega^3|\nu(\omega)|^2$$

$|\nu(\omega)|^2$ is the proportional to the intensity spectrum of the squeezed vacuum. For a monochromatic squeezed vacuum state, Ω, μ and ν are given by

$$\Omega = 2\omega_0$$

$$\mu(\omega) = \delta(\omega - \omega_0)\cosh(r) \tag{I.12}$$

$$\nu(\omega) = -\delta(\omega - \omega_0)\sinh(r)$$

Hence,

$$U^\dagger a(\omega)U = \delta(\omega - \omega_0)\cosh(r)\,a(\omega) - \delta(\omega - \omega_0)\sinh(r)\,a^\dagger(2\Omega - \omega)$$

$$U^\dagger a^\dagger(\omega)U = \delta(\omega - \omega_0)\cosh(r)\,a^\dagger(\omega) - \delta(\omega - \omega_0)\sinh(r)\,a(2\Omega - \omega)$$

$$|\cosh(r)|^2 - |\sinh(r)|^2 = 1 \tag{I.13}$$

$$\langle a(\omega)a(\omega')\rangle = -\delta(\omega - \omega_0)\delta(\omega' - \omega_0)\cosh(r)\sinh(r)\,\delta(2\omega_0 - \omega - \omega')$$

$$\left\langle a(\omega)a^\dagger(\omega')\right\rangle = \delta(\omega - \omega_0)\delta(\omega' - \omega_0)\cosh^2(r)\,\delta(\omega - \omega')$$

$$\left\langle a^\dagger(\omega)a(\omega')\right\rangle = \delta(\omega - \omega_0)\delta(\omega' - \omega_0)\sinh^2(r)\,\delta(\omega - \omega')$$

$$\langle a^\dagger(\omega)a^\dagger(\omega')\rangle = -\delta(\omega-\omega_0)\delta(\omega'-\omega_0)\cosh(r)\sinh(r)\,\delta(2\omega_0-\omega-\omega')$$

We now consider the 1st contribution to the energy $\langle \boldsymbol{P},\{0_{\boldsymbol{k},j}\}|U^\dagger V_1 U|\boldsymbol{P},\{0_{\boldsymbol{k},j}\}\rangle$:

$$\begin{aligned}&\langle \boldsymbol{P},\{0_{k,j}\}|U^\dagger V_1 U|\boldsymbol{P},\{0_{k,j}\}\rangle \\ &= \frac{e}{m}\sum_{\boldsymbol{p},k_j}\sqrt{\frac{2\pi\hbar}{V\omega_k}}\,\langle \boldsymbol{P},\{0_{k,j}\}|\begin{pmatrix} e_{\boldsymbol{k}j}\cdot \boldsymbol{p}c^\dagger_{\boldsymbol{p}+\hbar\boldsymbol{k}}c_p\left(\mu(\omega)a_{(\boldsymbol{k}_j)}+\nu(\omega)a^\dagger_{(2\mathbf{k_p}-\boldsymbol{k}_j)}\right) \\ +e^*_{\boldsymbol{k}j}\cdot\boldsymbol{p}\left(\mu^*(\omega)a^\dagger_{(\boldsymbol{k}_j)}+\nu^*(\omega)a_{(2\mathbf{k_p}-\boldsymbol{k}_j)}\right)c^\dagger_{\boldsymbol{p}-\hbar\boldsymbol{k}}c_p\end{pmatrix}|\boldsymbol{P},\{0_{k,j}\}\rangle = 0\end{aligned} \quad \text{(I.14)}$$

The 2nd contribution to the energy is

$$\begin{aligned}&\langle \boldsymbol{P},\{0_{k,j}\}|U^\dagger V_2 U|\boldsymbol{P},\{0_{k,j}\}\rangle \\ &\quad= \frac{\pi\hbar e^2}{mV}\sum_{\boldsymbol{pp'}}\sum_{\boldsymbol{kk'},jj'}\frac{1}{\sqrt{\omega_k\omega_{k'}}} \\ &\quad\times\Big(e_{\boldsymbol{k}j}\cdot e_{\boldsymbol{k'}j'}\delta_{\boldsymbol{p},\boldsymbol{p'}}\left(\mu(\boldsymbol{k}j)\nu(\boldsymbol{k'}j')\delta\big(2\mathbf{k_p}-\boldsymbol{k}j-\boldsymbol{k'}j'\big)\right)\delta_{\boldsymbol{p}-\boldsymbol{p'},\hbar(\boldsymbol{k}+\boldsymbol{k'})}+e_{\boldsymbol{k}j} \\ &\quad\cdot e^*_{\boldsymbol{k'}j'}\delta_{\boldsymbol{p},\boldsymbol{p'}}\,\mu(\boldsymbol{k}j)\mu^*(\boldsymbol{k'}j')\delta(\boldsymbol{k}j-\boldsymbol{k'}j')\delta_{p-p',\hbar(k-k')}-\frac{1}{2}c^\dagger_{\boldsymbol{p}}c_{\boldsymbol{p}}\delta_{\boldsymbol{pp'}}\delta_{jj'} \\ &\quad+h.c.\Big) \\ &= \frac{\pi\hbar e^2}{mV}\sum_{\boldsymbol{k},j}\left(\frac{1}{\omega_{\boldsymbol{k}j}}(2|\mu(\boldsymbol{k}j)|^2-1)\right) = \frac{\pi\hbar e^2}{mV}\sum_{\boldsymbol{k},j}\left(\frac{1}{\omega_{\boldsymbol{k}j}}(2|\mu(\boldsymbol{k}j)|^2-1)\right)\end{aligned}$$

Changing the sum to an integral, the term becomes:

$$\langle \boldsymbol{P},\{0_{k,j}\}|U^\dagger V_2 U|\boldsymbol{P},\{0_{k,j}\}\rangle = \frac{\hbar e^2}{8\pi^2 m}\sum_j\int d^3\boldsymbol{k}_j\left(\frac{1}{\omega_{\boldsymbol{k}j}}\left(2\left|\mu(\boldsymbol{k}_j)\right|^2-1\right)\right)$$

The energy shift by squeezing is found by the substraction:

$$\begin{aligned}U_p^{(\text{q,multimode})} &\equiv \langle \boldsymbol{P},\{0_{k,j}\}|U^\dagger V_2 U|\boldsymbol{P},\{0_{k,j}\}\rangle - \langle \boldsymbol{P},\{0_{k,j}\}|V_2|\boldsymbol{P},\{0_{k,j}\}\rangle = \\ &= \frac{\hbar e^2}{4\pi^2 m}\sum_j\int d^3\boldsymbol{k}_j\,\frac{\left|\nu(\boldsymbol{k}_j)\right|^2}{\omega_{\boldsymbol{k}j}}\end{aligned} \quad \text{(I.15)}$$

This term is a generalization of the pondermotive energy well known from the case of coherent state. To see this explicitly, let us assume that the squeezed vacuum field is single mode, so that $|\nu(\boldsymbol{k})| = 0$ for any $\boldsymbol{k}\neq\boldsymbol{k}_p$ and $\left|\mu(\boldsymbol{k_p})\right|^{\mathbf{2}} = \cosh^2\left(r_{\boldsymbol{k_p}}\right); \left|\nu(\boldsymbol{k_p})\right|^{\mathbf{2}} = \sinh^2\left(r_{\boldsymbol{k_p}}\right) = N_{SV}$. Then, the energy shift of this term is

$$\langle \boldsymbol{P}, \{0_{k,j}\} | U^\dagger V_2 U | \boldsymbol{P}, \{0_{k,j}\} \rangle - \langle \boldsymbol{P}, \{0_{k,j}\} | V_2 | \boldsymbol{P}, \{0_{k,j}\} \rangle$$
$$= \frac{\pi \hbar e^2}{mV} \frac{1}{\omega_{\boldsymbol{k}_p}} \left(\left(2\cosh(\boldsymbol{k_p})^2 - 1 \right) - (2-1) \right) \tag{I.16}$$
$$= \frac{\pi \hbar e^2}{mV} \frac{2}{\omega_{\boldsymbol{k}_p}} \sinh(\boldsymbol{k_p})^2 = \frac{\pi \hbar e^2}{mV} \frac{2}{\omega_{\boldsymbol{k}_p}} N_{SV}$$

The intensity of the squeezed vacuum beam is

$$I_{\text{vac}} = c\hbar\omega \frac{N_{SV}}{V} \tag{I.17}$$

Therefore,

$$\langle \boldsymbol{P}, \{0_{k,j}\} | U^\dagger V_2 U | \boldsymbol{P}, \{0_{k,j}\} \rangle - \langle \boldsymbol{P}, \{0_{k,j}\} | V_2 | \boldsymbol{P}, \{0_{k,j}\} \rangle = \frac{\pi \hbar e^2}{mV} \frac{2}{\omega_{\boldsymbol{k}_p}} \frac{V I_{\text{vac}}}{c\hbar\omega_{\boldsymbol{k_p}}}$$
$$= \frac{8\pi e^2}{mc} \frac{I_{\text{vac}}}{4\omega_{\boldsymbol{k_p}}^2} = \frac{2e^2}{m\epsilon_0 c} \frac{I_{\text{vac}}}{4\omega_{\boldsymbol{k_p}}^2} \equiv U_p^{(q)} \tag{I.18}$$

This is the squeezed vacuum analogue to the pondermotive energy, as discussed in the main text.

The third and last contribution to the energy will result in a squeezing dependent mass shift. After replacing the sum over $\boldsymbol{k}$ with an integral, it is given by

$$\langle \phi | U^\dagger V_1 \frac{1}{E_0 - H_0} (1 - P_0) V_1 U | \phi \rangle =$$
$$\frac{2\pi e^2 \hbar}{4\pi^2 m^2} \sum_j \int d^2\boldsymbol{k}_j \frac{1}{\omega_k} |e_{\boldsymbol{k}j}$$
$$\cdot \boldsymbol{P}|^2 \left\{ \frac{|\nu(\boldsymbol{k_j})|^2}{\frac{-\boldsymbol{P} \cdot \hbar \boldsymbol{k_j}}{m} - \hbar(2\omega_p - \omega_j)\left(|\mu(2\mathbf{k}_p - \boldsymbol{k_j})|^2 + |\nu(2\mathbf{k}_p - \boldsymbol{k_j})|^2 \right)} + \atop + \frac{|\mu(\boldsymbol{k_j})|^2}{\frac{\boldsymbol{P} \cdot \hbar \boldsymbol{k_j}}{m} - \hbar\omega_j \left(|\boldsymbol{\mu}(\boldsymbol{k_j})|^2 + |\nu(\boldsymbol{k_j})|^2 \right)} \right\} \tag{I.19}$$

Single mode squeezed vacuum in a cavity of volume V

For a single mode squeezed vacuum in a cavity of volume V, the energy is

$$E(r) = \underbrace{\frac{P^2}{2m} + \frac{2\pi e^2\hbar}{m^2 V}\sum_{k_j} \frac{\left|e_{\boldsymbol{k}j}\cdot\boldsymbol{P}\right|^2}{\omega_k}\left\{\frac{1}{\frac{\boldsymbol{P}\cdot\hbar\boldsymbol{k_j}}{m} - \hbar\omega_j}\right\}}_{\frac{P^2}{2m_e} = \frac{P^2}{2(m+\delta m)}} + U_{\mathrm{p}}^{(\mathrm{q})}$$

$$+\frac{2\pi e^2\hbar}{m^2 V}\frac{\sin^2\!\left(\theta_{\boldsymbol{P},\boldsymbol{k}}\right)}{\omega_k}|\boldsymbol{P}|^2\left(\frac{\left(\frac{\boldsymbol{P}\cdot\hbar\boldsymbol{k}}{m} + \hbar\omega\cosh^2(2r)\right)}{\left(\frac{\boldsymbol{P}\cdot\hbar\boldsymbol{k}}{m}\right)^2 - (\hbar\omega\cosh(2r))^2} - \frac{1}{\frac{\boldsymbol{P}\cdot\hbar\boldsymbol{k_j}}{m} - \hbar\omega_j}\right) = \frac{P^2}{2m^*(r)} + U_{\mathrm{p}}^{(\mathrm{q})} \tag{I.20}$$

Where we have used $\left|e_{\boldsymbol{k}j}\cdot\boldsymbol{P}\right|^2 = P^2\, sin^2\left(\theta_{\boldsymbol{k_j}}\right)$. Note that e_{kj} is a polarization vector and θ_{k_j} is the angle of the wavevector, such that at $\theta_{k_j} = 0$ we have $\boldsymbol{P}\cdot e_{\boldsymbol{k}_j} = 0$ and the polarization is parallel with $\boldsymbol{k_j}$. The renormalized mass is given by:

$$\frac{1}{m^*(r)} = \frac{1}{m_e} + \frac{4\pi e^2\hbar}{m^2 V}\frac{\sin^2\!\left(\theta_{\boldsymbol{P},\boldsymbol{k}}\right)}{\omega_k}\left(\frac{\left(\frac{\boldsymbol{P}\cdot\hbar\boldsymbol{k}}{m} + \hbar\omega\cosh^2(2r)\right)}{\left(\frac{\boldsymbol{P}\cdot\hbar\boldsymbol{k}}{m}\right)^2 - (\hbar\omega\cosh(2r))^2} - \frac{1}{\frac{\boldsymbol{P}\cdot\hbar\boldsymbol{k_j}}{m} - \hbar\omega_j}\right) \tag{I.21}$$

Moving to atomic units, we set $\hbar = 1$, $m_e = 1$, $m = 1 - \delta m$, $e = 1$:

$$E = \frac{P^2}{2m^*(r)} = \frac{P^2}{2}\left(1 + \frac{4\pi}{m^2 V}\frac{\sin^2\!\left(\theta_{\boldsymbol{P},\boldsymbol{k}}\right)}{\omega_k}\left(\frac{\left(\frac{\boldsymbol{P}\cdot\boldsymbol{k}}{m} + \omega\cosh^2(2r)\right)}{\left(\frac{\boldsymbol{P}\cdot\boldsymbol{k}}{m}\right)^2 - (\omega\cosh(2r))^2} - \frac{1}{\frac{\boldsymbol{P}\cdot\boldsymbol{k}}{m} - \omega}\right)\right) \tag{I.22}$$

This translates to the renormalized mass of a free electron coupled to a single mode of squeezed vacuum in a cavity of volume V:

$$m^*(r) = 1 - \frac{4\pi(1+2\delta m)}{V}\frac{\sin^2(\theta_{\boldsymbol{P},\boldsymbol{k}})}{\omega_k}\left(\frac{(\boldsymbol{P}\cdot\boldsymbol{k}(1+\delta m) + \omega\cosh^2(2r))}{\left(\boldsymbol{P}\cdot\boldsymbol{k}(1+\delta m)\right)^2 - (\omega\cosh(2r))^2} - \frac{1}{\boldsymbol{P}\cdot\boldsymbol{k}(1+\delta m) - \omega}\right) \tag{I.23}$$

Notably, the mass renormalization is equal to 1 if $\theta_{\boldsymbol{P},\boldsymbol{k}} = 0$ and $\theta_{\boldsymbol{P},\boldsymbol{k}} = \frac{\pi}{2}$. Mass renormalization is maximized at $\theta_{\boldsymbol{P},\boldsymbol{k}} = \pi/4$, which results in:

$$m^*_{\left(\theta_{\boldsymbol{P},\boldsymbol{k}}=\frac{\pi}{4}\right)}(r) = 1 - \frac{2\pi(1+2\delta m)}{\omega_k V}\left(\frac{Pk(1+\delta m)/\sqrt{2} + \omega\cosh^2(2r)}{\left(Pk(1+\delta m)/\sqrt{2}\right)^2 - \omega^2\cosh^2(2r)} - \frac{1}{Pk(1+\delta m)/\sqrt{2} - \omega}\right) \tag{I.24}$$

Numerical evaluation of squeezing dependent mass renormalization in free-space geometries

We consider an electron with an undressed energy of $20eV$, interaction with a single mode of squeezed vacuum with a wavelength $800nm$. The interaction angle is $\theta_{\boldsymbol{P},\boldsymbol{k}} = \pi/4$. The intensity of the squeezed vacuum beam is $I = 2\times 10^{14}\, Watt/cm^2$. The single photon amplitude $\epsilon^{(1)} \equiv \sqrt{\hbar\omega/2\epsilon_0 V}$ is set to $5e-8a.u.$ With these parameters, we find that the correction to the mass is $\left|\frac{\delta m}{m}\right| \approx 10^{-15}$.

II. Numerical methodology

In this section, we describe the details of the numerical calculations presented in the main text.

Interaction picture representation: bound electron in single mode squeezed vacuum & single mode coherent states

We begin by considering interaction with squeezed vacuum. Without interaction between the atom and the light, the state of the system is stationary and equals to

$$|\psi\rangle = |\mathrm{g.s}\rangle|0,r\rangle \tag{II.1}$$

in which $|\mathrm{g.s}\rangle$ is the ground state of a model Xe atomic potential $V_a(x)$, and $|0,r\rangle$ is a squeezed vacuum state at frequency Ω. The model Xe atom is given by

$$V_a(x) = -0.63\exp(-0.1424x^2)$$
$$|0,r\rangle = \hat{S}(re^{i\theta})|0\rangle \tag{II.2}$$

Here, $\hat{S}(re^{i\theta})$ is a squeezing operator for the mode Ω, r squeezing parameter, θ squeezing angle. $|0\rangle$ is the vacuum mode of the radiation field. To solve for the time evolution of the interacting light matter system, we write their joint Hamiltonian as

$$\hat{H}(x,t) = -\frac{1}{2m}\partial_x^2 + V(x) - ex\hat{E}^{(\mathrm{q})} + \sum_n \hbar\Omega\left(\hat{a}_\Omega^\dagger\hat{a}_\Omega + \frac{1}{2}\right)$$
$$\hat{E}^{(\mathrm{q})} = i\epsilon^{(\mathrm{q})}\left(\hat{a}_\Omega - \hat{a}_\Omega^\dagger\right) \tag{II.3}$$
$$\epsilon^{(\mathrm{q})} = \sqrt{\frac{\hbar\Omega}{2\epsilon_0 V}}$$

in which $\epsilon^{(\mathrm{q})}$ is the "single-photon amplitude" and $\hat{a}_\Omega, \hat{a}_\Omega^\dagger$ are annihilation and creation operators for a mode Ω. Transitioning to the interaction picture with respect to the operator $\sum_n \hbar\Omega\left(\hat{a}_\Omega^\dagger\hat{a}_\Omega + \frac{1}{2}\right)$ we have $\hat{a}_\Omega \to \hat{a}_\Omega e^{-i\Omega t}$, and the Hamiltonian becomes:

$$\hat{H}(x,t) = -\frac{1}{2m}\partial_x^2 + V(x) - ex\hat{E}^{(q)}(t)$$
$$\hat{E}^{(\mathrm{q})} = i\epsilon^{(\mathrm{q})}\left(e^{-i\Omega t}\hat{a}_\Omega - e^{i\Omega t}\hat{a}_\Omega^\dagger\right) \tag{II.4}$$

We now transform the quantum state of light from an initial condition encoded in $|\psi\rangle$, to a parameter in the Hamiltonian. We move to a picture of $\tilde{H}, |\tilde{\psi}\rangle$ that are defined by:

$$|\tilde{\psi}\rangle = \hat{S}^\dagger(re^{i\theta})|\psi\rangle$$
$$\tilde{H} = \hat{S}^\dagger(re^{i\theta})\hat{H}\hat{S}(re^{i\theta}) \tag{II.5}$$

In this representation, the non-interacting photonic state of the system transforms from $|0,r\rangle$ to $|0\rangle$, the vacuum state. The creation and annihilation operators transform according to a Bogoliubov transformation:

$$\hat{a}_\Omega \to \hat{a}_\Omega\cosh(r) - e^{i\theta}\hat{a}_\Omega^\dagger\sinh(r)$$
$$\hat{a}_\Omega^\dagger \to \hat{a}_\Omega^\dagger\cosh(r) - e^{-i\theta}\hat{a}_\Omega\sinh(r) \tag{II.6}$$

The electric field operator transforms as

$$\hat{S}^\dagger(re^{i\theta})\hat{E}^{(\mathrm{q})}\hat{S}(re^{i\theta})$$
$$= i\epsilon^{(\mathrm{q})}\left(\cosh(r)\left(e^{-i\Omega t}\hat{a}_\Omega - e^{i\Omega t}\hat{a}_\Omega{}^\dagger\right) + \sinh(r)\left(e^{i\Omega t}\hat{a}_\Omega e^{-i\theta} - e^{-i\Omega t}\hat{a}_\Omega{}^\dagger e^{i\theta}\right)\right) \tag{II.7}$$

Therefore, within this picture, the Hamiltonian $\tilde{H}$ and uncoupled state $|\tilde{\psi}\rangle$ are given by

$$\widetilde{H}_{\mathrm{S.V}} = -\frac{1}{2m}\partial_x^2 + V(x) - i\epsilon^{(q)}ex\begin{pmatrix} e^{-i\Omega t}\left(\hat{a}_\Omega \cosh(r) - e^{i\theta}\hat{a}_\Omega{}^\dagger \sinh(r)\right) \\ -e^{i\Omega t}\left(\hat{a}_\Omega{}^\dagger \cosh(r) - e^{-i\theta}\hat{a}_\Omega \sinh(r)\right) \end{pmatrix} \quad \text{(II.8)}$$

In the case of an interaction with a coherent state, the uncoupled state of the system is given by $|\mathrm{g.s.}, \alpha\rangle$ where $|\alpha\rangle = D(\alpha)|0\rangle$ and $D(\alpha)$ is a coherent shift operator. For this case, we employ an interaction picture $|\tilde{\psi}\rangle = \widehat{D}^\dagger(\alpha)|\psi\rangle$, so that the uncoupled state becomes again $|\mathrm{g.s}\rangle|0\rangle$. In this frame the Hamiltonian becomes

$$\widetilde{H}_{\mathrm{CS}} = -\frac{1}{2m}\partial_x^2 + V(x) - ex\langle\alpha|\hat{E}_\Omega(t)|\alpha\rangle - i\epsilon^{(q)}ex\left(e^{-i\Omega t}(\hat{a}_\Omega) - e^{i\Omega t}(\hat{a}_\Omega{}^\dagger)\right) \quad \text{(II.9)}$$

Time evolution

To perform the time evolution for a free electron in squeezed vacuum or in a coherent state of light, we employ the Hamiltonians $\widetilde{H}_{\mathrm{S.V}}(t)$ and $\widetilde{H}_{\mathrm{CS}}(t)$ without the atomic potential $V_a(x)$. The initial electron state is given by a Gaussian $|g\rangle \propto \exp(-x/4\sigma_0^2)$. The light-matter system is initialized to $|g\rangle|0\rangle$ and time evolution under $\widetilde{H}_{\mathrm{S.V}}(t)$ and $\widetilde{H}_{\mathrm{CS}}(t)$ is implemented using the (t, t') method [24].

Numerical basis

For the numerical implementation of all algorithms[24], we represent the Floquet Hamiltonian as a tensor $[\mathcal{H}_f]_{(n'm'v'),(nmv)}$ where n, m, v are indices of temporal, spatial, and photonic basis functions, respectively:

$$|n) = \frac{1}{\sqrt{T}}e^{in\Omega t}\,; n = -N, \dots, -1,0,1, \dots, N_n$$

$$|m\rangle = \sqrt{\frac{2}{L}}\sin\left(\frac{\pi m(x + L/2)}{L}\right); m = 1,2,3, \dots, N_m \quad \text{(II.10)}$$

$$|v\rangle = \frac{\left(\hat{a}_\Omega^\dagger\right)^v}{\sqrt{v!}}|0\rangle; v = 0, \dots \mathrm{N}_v$$

Notably, $T = 2\pi/\Omega$, and L is the size of the numerical box, spanned from $x = -\frac{L}{2}$ to $x = \frac{L}{2}$. The different terms making up $[\mathcal{H}_f]_{(n'm'v'),(nmv)}$ are explicitly represented as:

$$\langle v'|(n'|\langle m'| - \frac{1}{2m}\partial_x^2|m\rangle|n)|v\rangle = \frac{\pi^2 m^2}{2L^2}\delta_{m,m'}\delta_{n,n'}\delta_{v,v'}$$

$$\langle v'|(n'|\langle m'|V_a(x)|m\rangle|n)|v\rangle = \langle m'|V_a(x)|m\rangle\delta_{n,n'}\delta_{v,v'} = V_a^{(m',m)}\delta_{n,n'}\delta_{v,v'} \quad \text{(II.11)}$$

$$\langle v'|(n'|\langle m'| - i\partial_t|m\rangle|n)|v\rangle = \delta_{v,v'}\delta_{m,m'}\delta_{n,n'}n\Omega$$

$$(n'|\cos(\Omega t + \phi)|n) = \left(\delta_{n',n-1}\frac{e^{-i\phi}}{2} + \delta_{n',n+1}\frac{e^{i\phi}}{2}\right)$$

$$\langle m'|x|m\rangle = \frac{4\left(-1+(-1)^{m+m'}\right)L\, m\, m'}{\pi^2(m'-m^2)^2}$$

$$\langle \nu'_\Omega|\hat{a}_\Omega|\nu_\Omega\rangle = \sqrt{\nu_\Omega}\delta_{\nu'_\Omega,\nu_\Omega-1}$$

$$\langle \nu'_\Omega|\hat{a}^\dagger_\Omega|\nu_\Omega\rangle = \sqrt{\nu_\Omega+1}\delta_{\nu'_\Omega,\nu_\Omega+1}$$

$$\left(n'\middle|e^{-i\Omega t}\middle|n\right) = (n'|n-1) = \delta_{n',n-1}$$

III. Fourier analysis of atomic dynamics in bright squeezed vacuum

In this section, we present a Fourier analysis of the bound state dynamics in Figure 3 of the main text.

Bound state dynamics

We consider the dynamics of the bound states, which are shown to undergo Rabi-like oscillations in Figure 3. (b) of the main text. The atomic system consists of three bound states of energies: $E_\mathrm{g} = -0.4451\ \mathrm{a.u.}$, $E_\mathrm{e} = -0.1400\ \mathrm{a.u.}$, and $E_\mathrm{e2} = -0.00014\ \mathrm{a.u.}$ The frequency of the driving field is $\Omega = 0.11\ \mathrm{a.u.}$, and the intensity of the pump is $I = 3.2 \times 10^{13}\ \mathrm{W\,cm^{-2}}$. Fourier analysis of the numerically obtained atomic inversion $\rho_\mathrm{ee}(t) - \rho_\mathrm{gg}(t)$ yields the frequency-domain atomic inversion $\rho_\mathrm{ee}(\omega) - \rho_\mathrm{gg}(\omega)$ (Figure S1).

The frequency domain atomic inversion shows two distinct spectral peaks. The lower frequency peak is at the detuning frequency between the pump and the transition frequency between the ground and the first excited state:

$$\delta_{12} = \mathrm{E_e} - \mathrm{E_g} - \Omega = (-0.14 + 0.4451) - 0.11 = 0.1951\ \mathrm{a.u.} \qquad \text{(III.1)}$$

This frequency corresponds to the frequency of Rabi-oscillations between the levels $|\mathrm{e}\rangle$ and $|\mathrm{g}\rangle$ in the highly detuned regime, as the generalized (detuned) Rabi-frequency is given by

$$\underbrace{\Omega_\mathrm{GR}}_{\text{Generalized Rabi}} = \sqrt{\underbrace{\delta}_{\text{detuning}}{}^2 + \underbrace{\Omega_R}_{\text{Resonant Rabi-frequency}}} \underbrace{\approx}_{\text{high detuning}} \delta \qquad \text{(III.2)}$$

The higher frequency peak is at the frequency component $0.44\ \mathrm{a.u} = \mathrm{E_{e2}} - E_g \approx I_p$, which is resonant with the 4'th harmonic of the driving field $4\Omega = 0.44\ \mathrm{a.u.}$

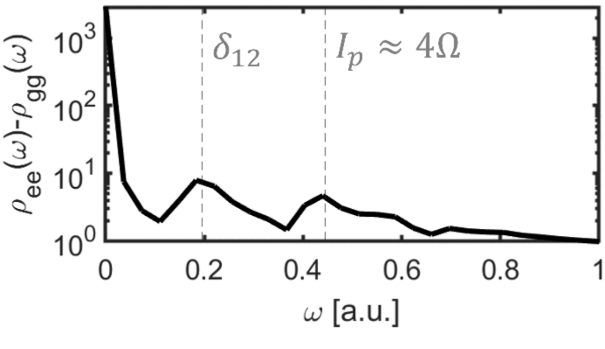

Figure S1: frequency domain atomic inversion, corresponding to the main text's Rabi-oscillations in Figure 3.(b). The atomic inversion displays two distinct peaks at the detuning frequency $\delta_{12} = E_e - E_g - \Omega$ and transition frequency between the ionization potential, which is resonant with the 4'th harmonic of the pump.

Sub-cycle dynamics of the width

We perform time-frequency analysis of the quantity $\Delta X^2(t)$ calculated for the model Xe atom driven by bright squeezed vacuum with an intensity $I = 3.2 \times 10^{13}\ \mathrm{W\,cm^{-2}}$. The time-

frequency spectrogram is defined by $G(\omega,\tau)=\int d\omega e^{-i\omega t}\Delta X^2(t)g(t,\tau)$ where $g(t,\tau)$ is a super-Gaussian window function $g(t,\tau)=\frac{1}{\Delta T}\exp\left(\left(-\frac{1}{2\Delta T^2}e^{-\frac{(t-\tau)^2}{2\Delta T^2}}\right)\right)$ with $\Delta T=0.25\,\mathrm{a.u.}$

The spectrogram $G(\omega,\tau)$ is presented in Figure S2, showing that high frequency component of the width occurs within a half-cycle temporal window, with a sub cycle structure corresponding to semi-classical recombination times (black overlay). This is consistent with the analogy between displacement trajectories in a coherent state and width trajectories in bright squeezed vacuum.

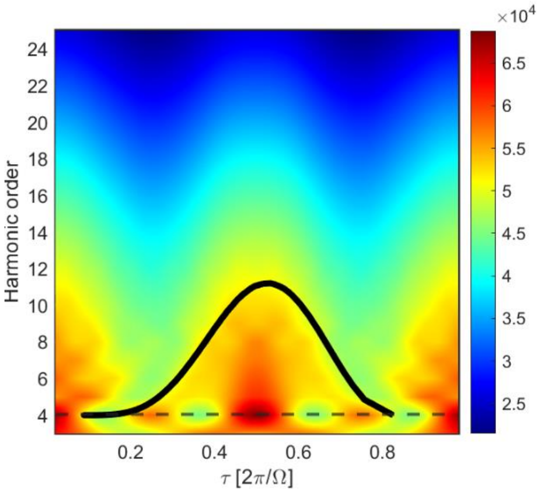

Figure S2: time-frequency analysis of the width $\Delta X^2(t)$ for an atomic wavepacket driven by bright squeezed vacuum with intensity $I=3.2\times10^{13}W\ cm^{-2}$. The ionization potential I_p is marked by a dashed line. High frequency component of the width occur within a half-cycle temporal window, with a sub cycle structure corresponding to semi-classical recombination times (black overlay). This is consistent with the analogy between displacement trajectories in a coherent state and width trajectories in bright squeezed vacuum.
